# Assessment of non-pharmacological nursing strategies for pain management in tumor patients: a systematic review and meta-analysis

**DOI:** 10.3389/fpain.2025.1447075

**Published:** 2025-04-15

**Authors:** Shen Yan, Feng Yan, Pei Liangyu, Xu Fei

**Affiliations:** Department of Cancer V, Longhua Hospital, Shanghai University of Traditional Chinese Medicine, Shanghai, China

**Keywords:** aromatherapy, massage, cancer-related pain, reflexology, acupuncture, acupressure

## Abstract

**Summary background:**

Cancer is a multifactorial disease associated with intense pain and fatigue. Pain is the main discomfort experienced during cancer treatment, particularly as a major side effect of chemotherapy.

**Objective:**

This study has aimed to investigate the effectiveness of non-pharmacological nursing strategies, including reflexology, aromatherapy, acupressure, massage therapy and acupuncture, in the management of cancer-associated pain. Moreover, it provides evidence-based recommendations for integrating these interventions into standard pain management protocols.

**Search methodology:**

We gathered data from three major online databases; PubMed, the Cochrane Library and Embase. For the analysis, we exclusively targeted randomized controlled trials (RCTs) assessing the effectiveness of non-pharmacological interventions in managing cancer-related pain. No language restrictions were applied, and pain was considered the primary outcome measure.

**Results:**

Seventeen RCTs (*n* = 1,070) were included in this meta-analysis from 166 eligible studies. The pooled effect size demonstrated that all evaluated non-pharmacological nursing strategies, including aromatherapy, massage, reflexology, acupressure and acupuncture significantly reduced cancer-related pain compared to usual care (*p* < 0.001). Moreover, the reflexology and massage showed negligible heterogeneity among other interventions.

**Conclusion:**

This meta-analysis found the significant effectiveness of non-pharmacological nursing strategies, particularly reflexology and massage in reducing cancer-related pain. The findings support their integration into clinical practice, providing evidence-based recommendations for enhancing standard pain management protocols.

## 1 Introduction

In cancer, pain is a severe discomfort and pain management is a highly critical aspect of patient care for cancer survivors. Comprehensive strategies are based on the patient’s condition, pain severity and disease pathogenesis (1). Despite the advancements and novelty in the healthcare system, the optimization of pain management remains a challenging step. However, nursing strategies for pain assessment and management play an important role in the survival of cancer survivors (2–4). We aimed to evaluate the effectiveness of non-pharmacological nursing interventions for the pain management among tumor patients in this systematic meta-analysis.

Cancer-related pain involves a complex interplay of psychological, physiological and social factors that ultimately have a significant impact on patients’ quality of life and the healthcare system (5). According to the World Health Organization (WHO), effective pain management is a fundamental human right, which emphasizes the importance of comprehensive approaches to alleviate suffering and improve quality of life (6). Nurses are frontline healthcare providers who play a significant role in pain assessment, therapeutic intervention and monitoring. Moreover, nurses can develop therapeutically effective strategies that are important for tumor-related pain management (7).

In the cancer care, pain management involves both pharmacological and non-pharmacological nursing approaches. In the pharmacological approach, analgesics are administered via several routes as prescribed by the oncologist. while, the therapeutic effectiveness of the prescribed analgesic is monitored and the dosage is adjusted or the medications are switched as needed (8, 9). In case of non-pharmacological interventions aromatherapy, massage, reflexology, acupressure and acupuncture are included (10). In this study, we gathered data on non-pharmacological strategies to evaluate their effectiveness in managing cancer-related pain.

Aromatherapy is a widely recognized therapeutic intervention for pain management. Moreover, several studies have demonstrated its effectiveness in reducing cancer-related pain. It is administered through inhalation, massage, or, in some cases, oral administration under professional supervision. Notably, the combination of aromatherapy with massage is widely practiced and has been proven effective for pain alleviation, as massage with essential oils is frequently used to reduce discomfort in cancer patients (11). In reflexology, pressure is applied to specific reflex points on the feet or hands to induce relaxation and promote healing. Foot reflexology is a highly practiced nursing strategy for alleviating pain in cancer patients (12). Similarly, acupressure, an ancient healing technique, offers potential relief for cancer-related pain. In this intervention, pressure is applied to specific points on the body, which stimulates the body’s natural healing abilities and promotes relaxation. Investigational studies revealed the significant effectiveness of acupressure for pain, anxiety and quality of life management among cancer patients (13). Furthermore, acupuncture is a traditional Chinese therapy in which the energy flow is rebalanced by inserting thin needles into specific points on the body, which ultimately promotes healing. Individual responses may vary; many individuals find relief from symptoms and experience improved quality of life through this holistic approach (14, 15).

Given the growing interest in non-pharmacological interventions, this study hypothesizes that integrating evidence-based non-pharmacological nursing strategies, including aromatherapy, massage, acupressure, acupuncture and reflexology can significantly enhance pain management outcomes in cancer patients.

## 2 Materials and methods

### 2.1 Literature search and search strategy

**Figure 1:**
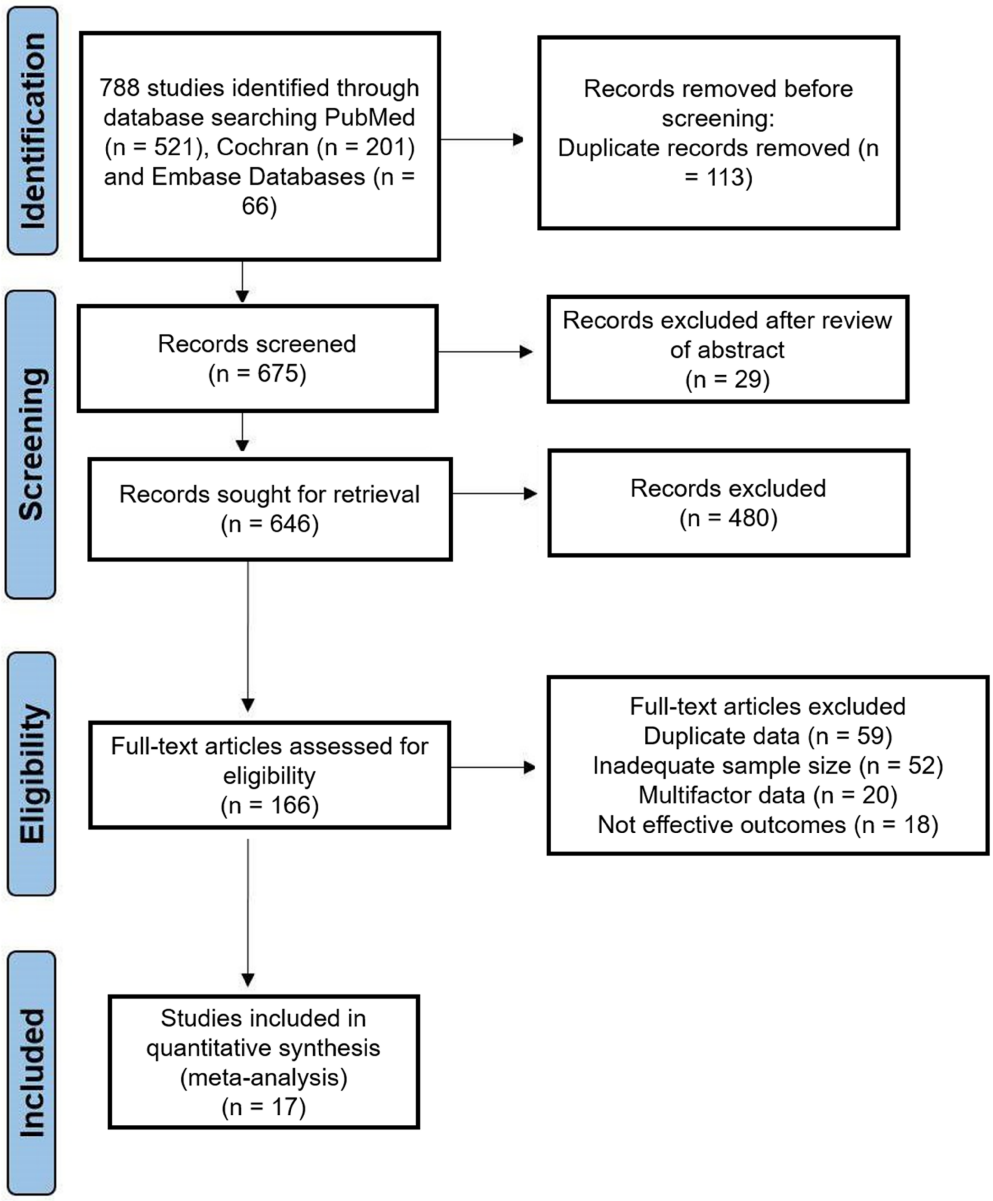
PRISMA flow diagram illustrating the study selection process, including the identification, screening, eligibility assessment and final inclusion of studies for meta-analysis.

During April–May 2024, we extracted relevant data from three databases, namely, the Cochrane Library, PubMed, and Embase. The relevant potential studies between 1990 and 2023 were included in this meta-analysis. For the data search, several Medical Subject Headings (MeSH) terms were used: “aromatherapy”, “cancer-related pain”, “cancer or malignancies”, “massage”, “reflexology”, “acupressure” and “acupuncture”. We included the data from search databases without language limitations. The study selection process is illustrated in the PRISMA flow chart (Figure 1).

### 2.2 Inclusion and exclusion criteria

The following inclusion criteria were considered for the data retrieval and measuring effects: (1) the targeted population included humans, (2) the study design was randomized controlled trials, (3) the population included adult males and females of all age groups, (4) only cancer patients were included, (5) research studies with sufficient sample sizes, based on statistical guidelines and previous research, were included to ensure reliable findings, (6) the experimental group received aromatherapy or massage or reflexology or acupressure or acupuncture to relieve pain, while the control group received usual care, (7) data from various periods were included to ensure complete coverage of the available literature, and (8) studies with robust statistical analyses, validated findings and clearly defined methodologies were included.

During the study selection process, to uphold the exclusion criteria, the following points were considered: (1) studies with unpublished data were restricted, (2) animal trials were excluded, (3) articles lacking proper methodology and outcomes were eliminated, and (4) derivative data sources such as review articles were limited.

### 2.3 Data analysis and validity assessment

To ensure the data accuracy and reliability, two independent reviewers screened and extracted the data from each study. Any discrepancies were resolved via the consultation with a third reviewer and no language restrictions were imposed. We compiled the extracted standardized data into a Microsoft Excel spreadsheet, creating a comprehensive database for our meta-analysis. The characteristics of the selected research studies are summarized in Table 1, which includes the following points: first author, publication year, country of publication, population, population size, participant types, age, pain management intervention and duration of treatment, measured outcomes and treatment evaluation.

**Table 1:** Summary of the included studies on non-pharmacological nursing intervention in the pain alleviation in cancer patients including study characteristics.

Sr. no.	Author/year	Country	Study type	Sample size	Tumor	Treatment strategy	Pain management interventions	Measured outcomes	Patients’ evaluation with pain management	Ref.
1	Wilkie et al./2000	USA	Randomized controlled trial	29 patients (14 control and 15 cancerous subjects)	Not specified	Massage	8 sessions of massage with aromatic oils (50 min per session)	PAT or SNVR	Massage relieved the pain associated with cancer	(34)
2	Weinrich et al./1990	USA	Randomized controlled trial	28 patients receiving chemotherapy and radiotherapy (14 control and 14 cancerous subjects)	Not specified	Swedish massage	10 min massage to the back	VAS	Massage was found a short-term strategy to relieve pain and showed significant outcomes in males than females	(24)
3	Wilkinson et al./2007	UK	Multicentered Randomized controlled trial	231 patients (115 control and 116 cancerous subjects)	Not specified	Aromatherapy massage with 20 essential oils	10 weeks of massage session	EORTC	After 10 weeks of the aromatherapy massage, the intensity of pain reduced but the outcomes were not strongly significant	(30)
4	Jane et al./2011	Taiwan	Randomized controlled trial	72 patients (36 control and 36 cancerous subjects)	Bone metastasis	Massage therapy	104 massage sessions; each one of 45 min.	PPI-VAS	Results found that massage therapy has positive effects on pain management in cancerous patients	(25)
5	Kim et al./2008	Korea	Randomized controlled trial	37 patients (18 control and 19 cancerous subjects)	Not specified	Foot reflexology	12 sessions	VAS	Foot reflexology in cancer patients improves pain significantly	(35)
6	Yayla et al./2019	Turkey	Randomized controlled trial	123 patients (41 control and 82 cancerous subjects)	Not specified	Inhalational aromatherapy with lavender oil and eucalyptus oil	3 drops of oil for 3 min	VAS	Aromatherapy with lavender oil has significant results than with eucalyptus oil	(20)
7	Soden et al./2004	UK	Randomized controlled trial	42 patients (13 control, 16 cancerous subjects received aromatherapy and 13 to the massage group)	Not specified	Aromatherapy with lavender oil and simple massage without lavender oil	–	VAS	Outcomes revealed that aromatherapy imparts slightly significant outcomes in the alleviation of cancer pain	(21)
8	Chang et al./2008	Korea	Randomized controlled trial	58 patients (30 control and 28 treatment subjects)	terminal cancer	Aromatherapy massage	–	VAS	Aroma hand massage significantly relieves the pain as compared to hand massage with general oil hand massage	(22)
9	Rambod et al./2019	Iran	Randomized controlled trial	72 patients (36 control and 36 treatment subjects)	Lymphoma	Foot reflexology	–	NRS	Reflexology improves the pain in lymphoma patients	(23)
10	Dikmen et al./2019	Turkey	Randomized controlled trial	80 patients (20 control and 20 in all three treatment subjects)	gynecologic cancer patients	Reflexology	–	BPI	Reflexology proved significant effect in the alleviation of pain	(36)
11	Hodgson et al./2012	USA	Randomized controlled trial	18 patients (9 control and 9 in the treatment group)	Solid tumor	Reflexology and Sweden massage	20 min sessions of both treatment	CNPI	Both interventions show significant outcomes in pain alleviation	(37)
12	Kim et al./2018	Korea	Randomized controlled trial	27 Advance staged cancer patients	Not specified	Intradermal acupuncture vs sham acupuncture	6 weeks trial	NRS	Intradermal acupuncture is a safe and effective treatment option for reducing the pain. However, the outcomes are insignificant in alleviation of pain from sham acupuncture treatment intervention	(27)
13	Bao et al./2013	USA	Randomized controlled trial	47 cancer patients	Breast cancer patients	Real acupuncture vs sham acupuncture	6 weeks trial	VAS	Results found insignificant outcomes in reducing pain scores while comparing sham and real acupuncture	(31)
14	Hershman et al./2018	USA	Randomized controlled trial	226 cancer patients	Early-stage breast cancer patients	Real acupuncture vs sham acupuncture vs control	30–45 min session for 6 weeks period (twice a week)	BPI-SF	Acupuncture proved a significant therapy to alleviate pain intensity	(26)
15	Yeh et al./2015	Taiwan	Randomized controlled trial	50 cancer patients	Not specified	Auricular point acupressure	1-week treatment duration	BPI	Outcomes proved that it is an effective technique for treating cancer-related pain	(28)
16	Castle et al./2023	USA	Randomized controlled trial	33 cancer patients	Not specified	Auricular point acupressure	3 days procedure; 3 times per day, each time press for 3 min	BPI	Acupressure proved an effective therapy to reduce pain in cancerous patients	(38)
17	Nia et al./2017	Iran	Randomized controlled trial	100 cancer patients	Not specified	Auricular point acupressure	12 acupressure	VAS	Outcomes have found that acupressure is a useful technique for relieving cancer-related pain	(29)

PAT, Pain Assessment Tool; SNVR, Skilled Nursing Visit Report form; VAS, Verbal Anchor and Midpoint Descriptors; EORTC, European Organization for Research and Treatment of Cancer; PPI-VAS, Present Pain Intensity-Visual Analogue Scale; NRS, Numerical pain scale; BPI, Brief Pain Inventory; CNPI, Checklist of Nonverbal Pain Indicators; BPI-SF, Brief Pain Inventory-Short Form.

For the analysis, we gathered the data and evaluated the validity across studies using a random-effects model, ensuring that the data were free from self-report bias. The extracted variables included sample sizes, means and standard deviations. Heterogeneity was quantified using the *I*^2^ statistic and assessed with Cochran’s *Q* test.

### 2.4 Risk of bias

**Figure 2:**
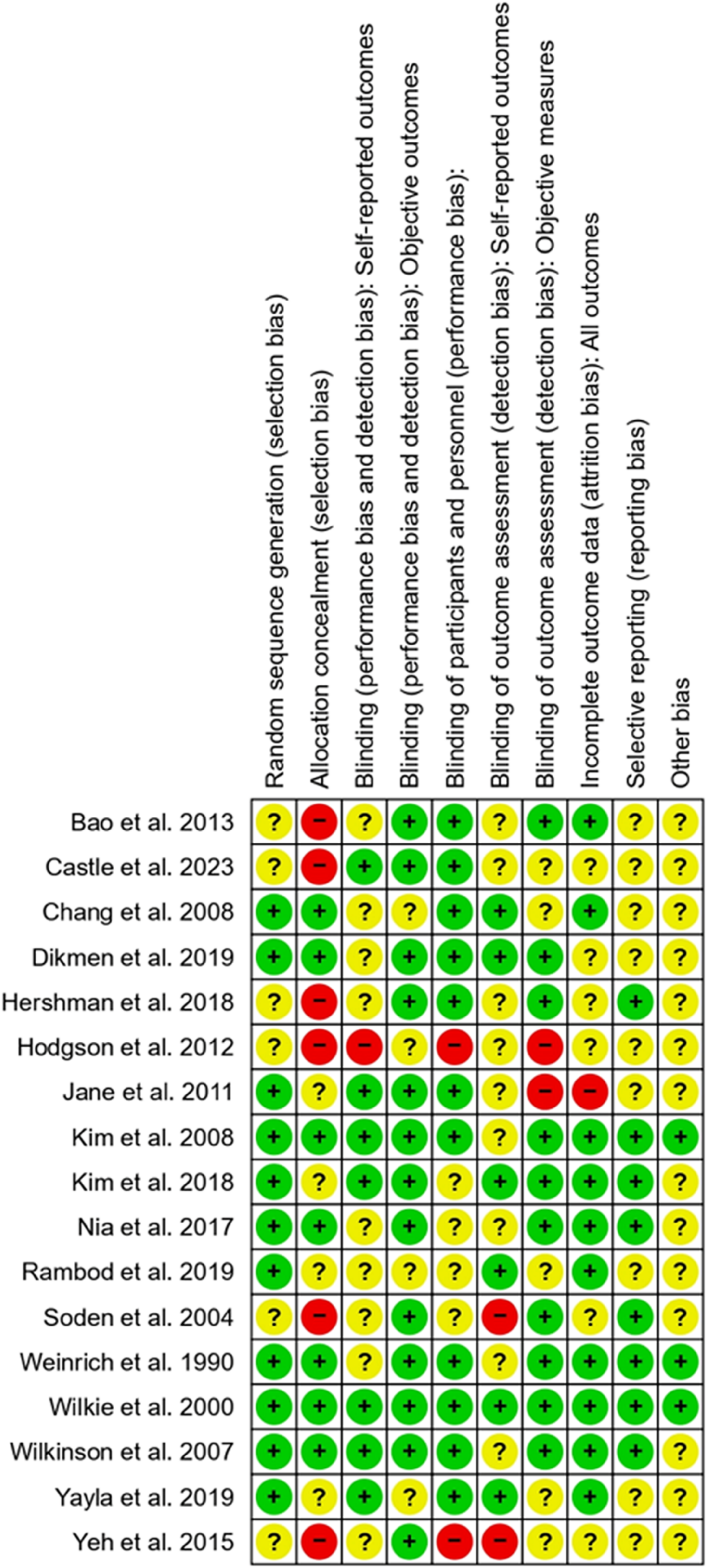
Risk of bias assessment for the included studies. The assessment was conducted using the Cochrane Risk of Bias Tool. Green circles (**+**) indicate a low risk of bias, yellow circles (?) represent an unclear risk of bias and red circles (−) denote a high risk of bias. This visual representation of outcomes provides an overview of the methodological quality and potential limitations of the studies included in the analysis.

We used the Cochrane Collaboration tool to evaluate the risk of bias in the included trials. This assessment covered the following domains: random sequence generation, allocation concealment, blinding of participants and personnel, blinding of outcome assessment and incomplete outcome data (attrition bias, referring to systematic differences between groups due to withdrawals leading to incomplete outcome data). The risk of bias assessment plot is visually summarized in Figure 2. Each domain is color-coded: green indicates low risk (+), yellow indicates unclear risk (?) and red indicates high risk (−). Most studies showed a mixture of low and unclear risks, with notably high risks in allocation concealment and blinding of participants and personnel. This comprehensive assessment highlights the variability in methodological quality among the included studies, providing a clear overview of potential biases impacting the meta-analysis.

### 2.5 Selectivity analysis

To ensure the accuracy and of our findings, we conducted a sensitivity analysis to assess the influence of multi-intervention studies on the meta-analysis results. Specifically, we examined the impact of the Hodgson et al. study on the pooled effect sizes of reflexology and massage therapy. However, in this study, reflexology and massage were administered separately with a washout period to minimize carryover effects and each intervention was assessed independently. This analysis aimed to determine whether the inclusion or exclusion of this study significantly altered the overall effect size. In a case, if results remain consistent with insignificant change in the overall effect size of analysis after excluding the study, confirming the robustness of the findings.

### 2.6 Statistical analysis

In this systematic meta-analysis, all the statistical analyses were performed using Review Manager, version 5.3 (Cochrane Collaboration, Oxford, England). We extracted the means, standard deviations (SDs) and sample sizes from the included studies. The meta-analysis was conducted in accordance with the PRISMA (Preferred Reporting Items for Systematic Reviews and Meta-Analyses) guidelines. We used a random effects model to estimate the effective mean size and pooled estimator of the continuous outcomes. To assess heterogeneity, *χ*^2^ and *I*^2^ inconsistency statistics were calculated. If the value of Cochran’s *Q* test was less than 0.10 (*p* < 0.10), heterogeneity was considered significant.

## 3 Results

### 3.1 Description of studies

This meta-analysis included 17 RCTs. Initially, we identified 788 studies related to pain alleviation techniques in cancer patients. After critical review, 166 RCTs were selected for the eligibility stage. Finally, after screening multifactorial data, inadequate assessment methods, etc., and removing duplicate publications, a total of 17 RCTs were selected for analysis. The study design for the meta-analysis is visually depicted in the PRISMA flow chart (Figure 1). These trials were published between 1990 and 2023, with sample sizes ranging from 18 to 231 patients. All of the included studies were randomized controlled trials.

### 3.2 Non-pharmacological strategies to alleviate cancer-related pain

#### 3.2.1 Aromatherapy

**Figure 3:**
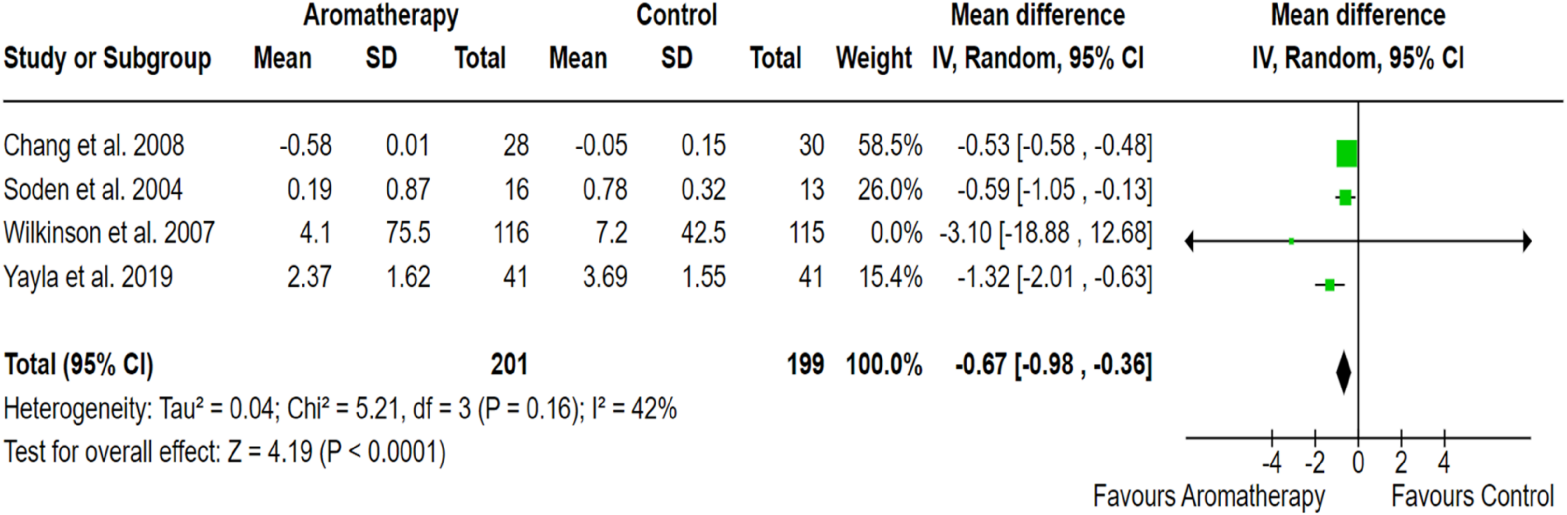
Forest plot showing the effect of aromatherapy on pain reduction compared to the control group. The *X*-axis represents the mean difference with a 95% confidence interval (CI), where negative values favor aromatherapy. Each study’s effect size is depicted as a green square, with the size proportional to its weight in the meta-analysis. The black diamond represents the overall pooled estimate. moderate heterogeneity (*I*^2^ = 42%) suggests variability in study design, sample sizes or intervention protocols. The overall effect (*p* < 0.00001) indicates a statistically significant benefit of aromatherapy.

Based on the 4 studies with 400 samples, aromatherapy for pain management in cancer patients was significantly more effective than usual care (SMD, −0.67; 95% CI, −0.98, −0.36; *p* < 0.001). Between-study heterogeneity was moderate (*I*^2^ = 42%). The pooled effect size of the outcome measures demonstrated the significant effectiveness of aromatherapy in alleviating cancer-related pain (Figure 3).

#### 3.2.2 Massage therapy

**Figure 4:**
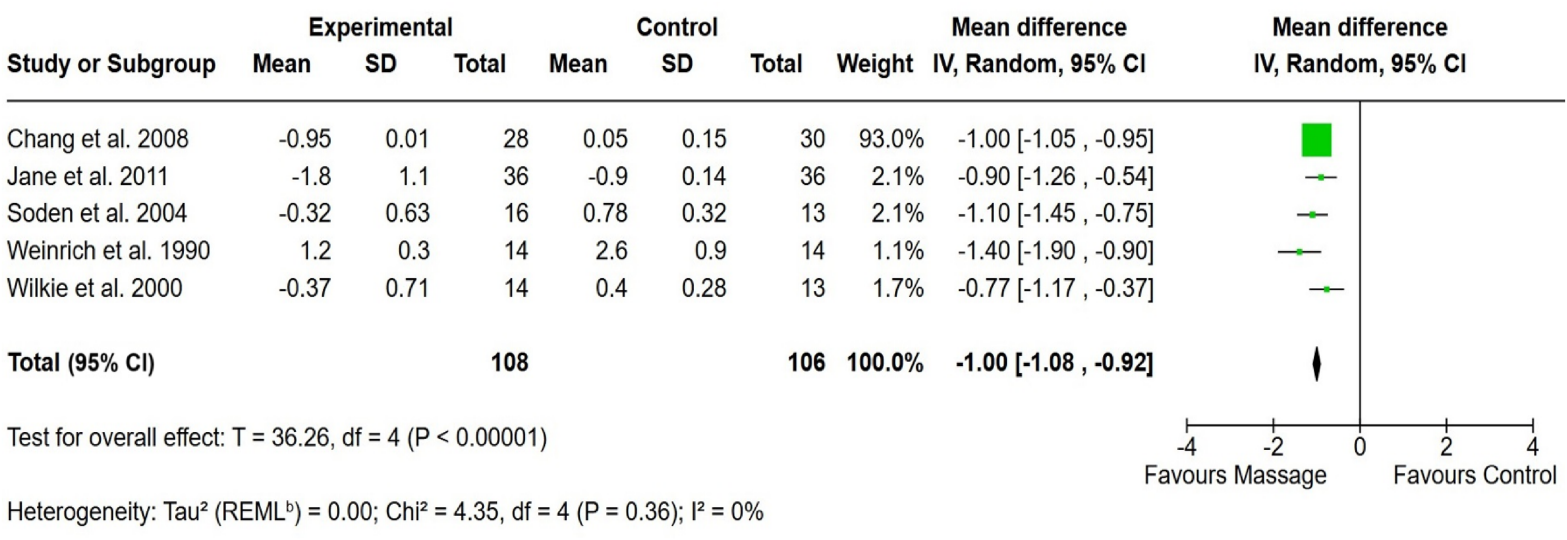
Forest plot showing the effect of massage on pain reduction compared to the control group. The *X*-axis represents the mean difference with a 95% confidence interval (CI), where negative values favor massage. Each study’s effect size is depicted as a green square, with the size proportional to its weight in the meta-analysis. The black diamond represents the overall pooled estimate. Low heterogeneity (*I*^2^ = 0%) suggests consistency across studies. The overall effect (*p* < 0.00001) indicates a statistically significant benefit of massage.

Massage therapy found significant effectiveness in pain management across 5 RCTs involving 215 cancer patients, compared to the control group receiving usual care (SMD: −0.1; 95% CI: −1.08 to −0.92; *p* < 0.001). Notably, between-study heterogeneity was negligible (0%), highlights the consistency and reliability of the results. Moreover, to assess the impact of including the Hodgson et al. study, a sensitivity analysis was performed, revealing a substantial change in effect size upon its exclusion. Given its significant influence on the pooled results, the study was removed from the massage therapy analysis to minimize heterogeneity and ensure a more accurate estimation of the intervention’s efficacy. The pooled effect size confirmed the substantial impact of massage therapy in alleviating cancer-related pain (Figure 4).

#### 3.2.3 Reflexology

**Figure 5:**
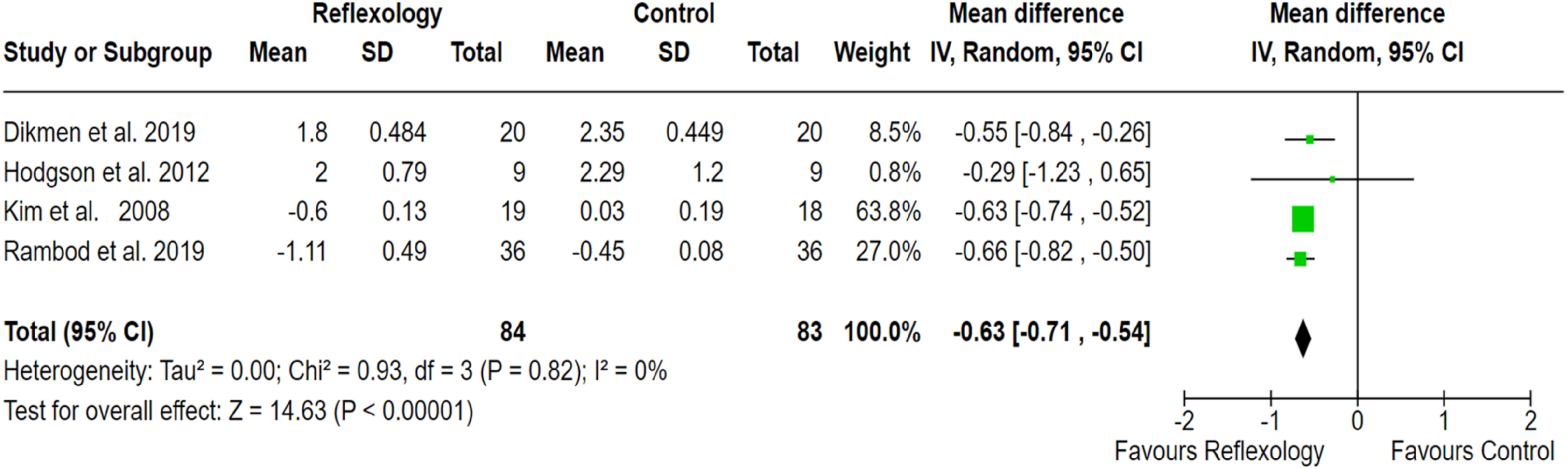
Forest plot showing the effect of reflexology on pain reduction compared to the control group. The *X*-axis represents the mean difference with a 95% confidence interval (CI), where negative values favor reflexology. Each study’s effect size is depicted as a green square, with the size proportional to its weight in the meta-analysis. The black diamond represents the overall pooled estimate. Low heterogeneity (*I*^2^ = 0%) suggests consistency across studies. The overall effect (*p* < 0.00001) indicates a statistically significant benefit of reflexology in pain alleviation.

Four clinical studies with a population size of 167 showed significant effectiveness in the management of cancer-related pain compared to usual care (SMD, −0.63; 95% CI, −1.71, −0.54; *p* < 0.001). Outcomes revealed no heterogeneity between studies (*I*^2^ = 0%). However, a sensitivity analysis was conducted to evaluate the impact of including data points from the Hodgson et al. study, as in this study combination intervention was employed. The analysis revealed no substantial differences in overall findings (effect size: −0.63, 95% CI: [−0.72, −0.55] vs. −0.63, 95% CI: [−0.71, −0.54]), confirming the robustness of the outcomes. The pooled effect size of the outcome measures revealed the significant effectiveness of reflexology in alleviating pain among patients with cancer (Figure 5).

#### 3.2.4 Acupressure and acupuncture

**Figure 6:**
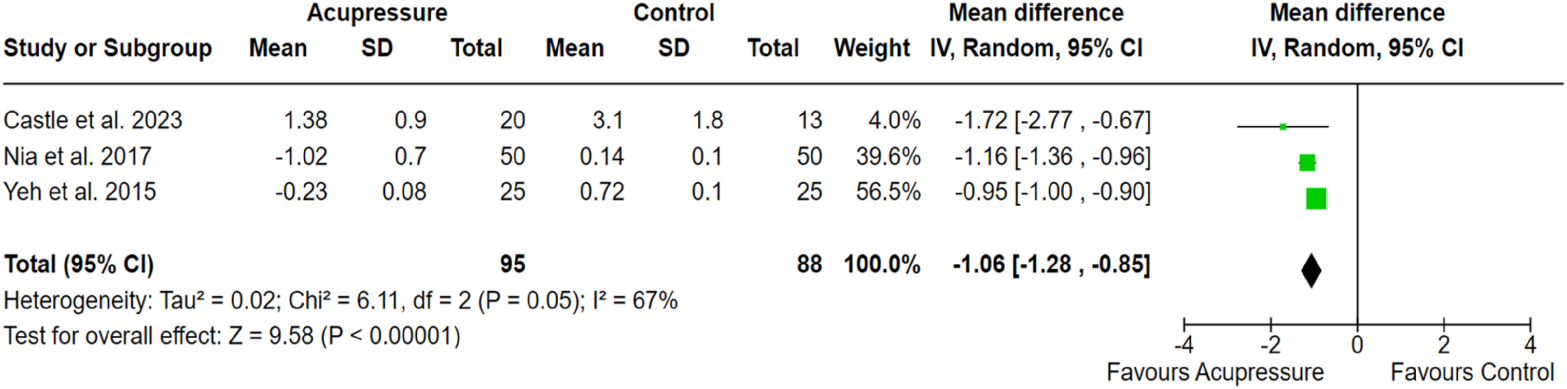
Forest plot showing the effect of acupressure on pain alleviation compared to the control group. The *X*-axis represents the mean difference with a 95% confidence interval (CI), where negative values favor acupressure. Each study’s effect size is shown as a green square, with the black diamond representing the overall pooled estimate. High heterogeneity (*I*^2^ = 67%) suggests variability in study design, sample sizes or intervention protocols. The overall effect (*p* < 0.00001) indicates a statistically significant benefit of acupressure.

**Figure 7:**
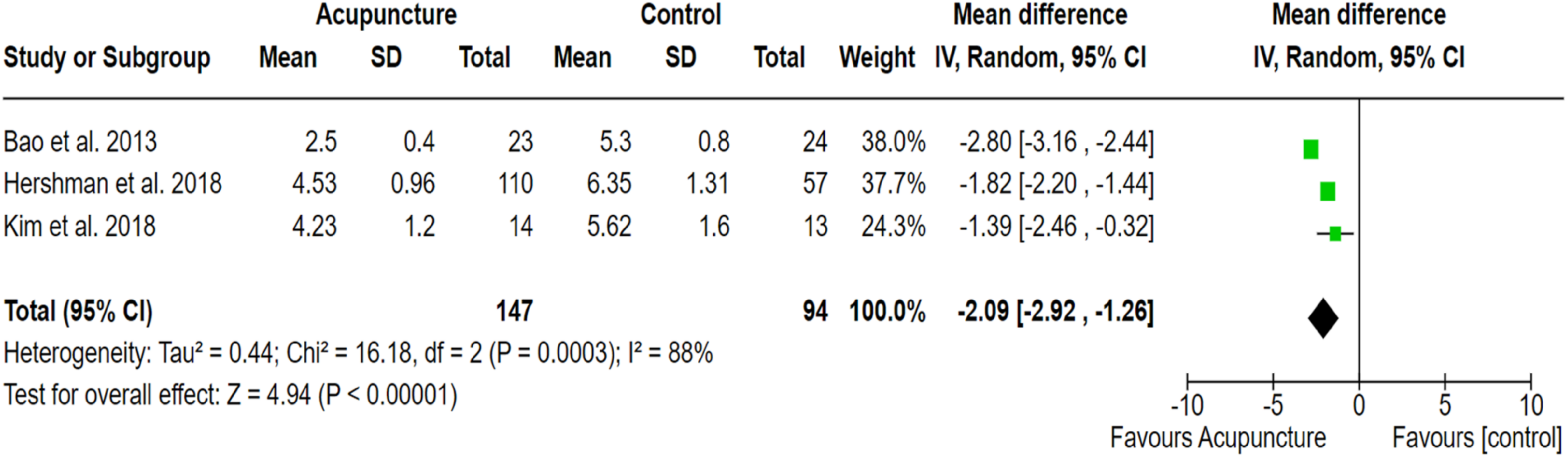
Forest plot showing the effect of acupuncture on pain alleviation compared to the control group. The *X*-axis represents the mean difference with a 95% confidence interval (CI), where negative values favor acupuncture. Each study’s effect size is shown as a green square, with the black diamond representing the overall pooled estimate. High heterogeneity (*I*^2^ = 88%) suggests variability in study design, sample sizes or intervention protocols. The overall effect (*p* < 0.00001) indicates a statistically significant benefit of acupuncture.

Three RCTs out of 17 clinical studies with a population size of 183 patients showed statistically significant effectiveness of acupressure (Figure 6). Similarly, three RCTs out of 17 studies with a sample size of 237 demonstrated the effectiveness of acupuncture in managing cancer-related pain compared to the control group receiving usual care (SMD, −1.06; 95% CI, −1.28, −0.85; *p* < 0.001; *I*^2^ = 67%; SMD, −2.09; 95% CI, −2.92, −1.26; *p* < 0.001; *I*^2^ = 88% respectively). The outcomes revealed moderate to high between-study heterogeneity. The pooled effect size of the outcome measures indicated the significant effectiveness of both acupressure and acupuncture in alleviating pain in cancer patients (Figures 6, 7).

## 4 Discussion

Cancer is a multifactorial disease associated with severe pain that affects both the physical and psychological well-being of patients (16). Cancer-related pain is often severe and uncontrolled, disrupting the emotional health of cancer patients and leading to increased depression and anxiety and a decline in functional and social activities (17). Therefore, effective pain management strategies are required to improve the quality of life of cancer survivors (16). Pain management strategies, including pharmacological interventions, like medication and non-pharmacological interventions, such as aromatherapy and acupressure (18). Nurses play an important role in managing cancer-related pain by providing continuous assessment, administering pain relief interventions and providing non-pharmacological interventions to improve patients’ quality of life. Their holistic approach ensures personalized care and effective pain management strategies (19).

This comprehensive meta-analysis included 17 randomized controlled clinical trials to demonstrate the effectiveness of frequently employed non-pharmacological nursing strategies in alleviating pain associated with cancer. We targeted five often used non-pharmacological interventions for analysis, namely, aromatherapy, massage therapy, reflexology, acupressure and acupuncture. According to our findings, all five interventions were significantly more effective at alleviating pain among cancer patients than among those in the control group receiving only usual care (*p* < 0.001). While all interventions demonstrated statistically significant effectiveness in managing cancer-related pain, some analyses exhibited moderate to high heterogeneity. Massage therapy and reflexology provided the most reliable evidence, with significant effect sizes (SMD: −0.1 and −0.63 respectively) and no between-study heterogeneity (*I*^2^ = 0%), ensuring consistency and low risk of bias. Aromatherapy (SMD: −0.67; *p* < 0.001) showed moderate heterogeneity (*I*^2^ = 42%), indicating some variability across studies. However, acupressure and acupuncture demonstrated the strongest effect sizes (SMD: −1.06 and −2.09 respectively), but their high heterogeneity (*I*^2^ = 67% and 88%) suggests the methodological inconsistencies and potential variability among studies. These findings highlight the effects of study design, sample size and outcome measures on effect estimates. Despite statistical significance across interventions, the robustness of massage therapy and reflexology findings makes them the most reliable approaches for pain relief in cancer patients. Further large-scale, high-quality trials are needed to validate the findings and minimize uncertainty in interventions with high heterogeneity.

Our results revealed the positive effects of aromatherapy on the management of cancer-related pain. Similarly, numerous studies have shown that aromatherapy has therapeutic benefits in alleviating pain for cancer patients (20–22). Similarly, consistent with our analysis, various investigational studies have revealed the effectiveness of reflexology (23), massage (24, 25), acupuncture (26, 27) and acupressure (28, 29) in alleviating pain associated with cancer.

However, some included studies reported insignificant outcomes, which may be attributed to various factors, including sample size, study design, intervention protocols and differences in patient populations. For instance, the RCTs on aromatherapy conducted by Wilkinson et al. (30) and Soden et al. (21) revealed a mild improvement in pain reduction, potentially due to variations in essential oil composition, duration of therapy or individual patient responses. Similarly, acupuncture trials, including Kim et al. (27) and Bao et al. (31) have reported insignificant outcomes, suggesting the possibility of a placebo effect or a need for refined methodologies in assessing acupuncture’s efficacy in pain management. These findings highlight the necessity for further large-scale, high-quality randomized controlled trials with standardized methodologies to confirm the effectiveness of non-pharmacological nursing interventions.

In 2011, a meta-analysis on the efficacy of acupuncture in the management of cancer-related pain was carried out by extracting data from three randomized clinical trials. Six databases were searched to extract relevant studies, and the outcomes revealed the effectiveness of acupuncture for pain alleviation among cancer patients (32). Similarly, a meta-analysis was carried out in 2011 to demonstrate the effectiveness of foot reflexology. The outcomes of that analysis revealed significant effectiveness in relieving pain and improving the quality of life in cancer patients (33).

The findings of our comprehensive meta-analysis suggest that non-pharmacological nursing strategies such as aromatherapy, massage therapy, reflexology, acupressure and acupuncture are significantly effective in alleviating cancer-related pain. The findings of our comprehensive meta-analysis suggest that non-pharmacological nursing strategies such as aromatherapy, massage therapy, reflexology, acupressure and acupuncture are significantly effective in alleviating cancer-related pain. However, moderate heterogeneity was observed due to the variation in sample sizes and outcome measures, the overall findings highlight the potential integration of these therapies into standard pain management protocols for cancer patients.

Our meta-analysis provides valuable new insights by providing evidence on the effectiveness of non-pharmacological nursing strategies for cancer-related pain management. By synthesizing data from multiple RCTs, this study strengthens the foundation for integrating these interventions into clinical practice. Despite the promising results, certain limitations must be acknowledged. The included studies exhibited variability in sample sizes and lacked subgroup analyses based on age, gender and type of cancer, which may affect the generalizability of our findings. Additionally, potential publication bias remains a concern, as studies with negative results are less likely to be published, potentially overestimating the effectiveness of these interventions. These factors necessitate the careful interpretation of the findings. To enhance the reliability of future research, standardized methodologies, diverse patient populations and long-term follow-ups should be prioritized. Further well-designed, large-scale RCTs are needed to validate these interventions and establish their sustained benefits in cancer pain management.

## 5 Conclusion

This meta-analysis highlights the effectiveness of non-pharmacological nursing strategies in alleviating cancer-related pain, offering valuable insights for clinical pain management. However, variations in sample sizes, intervention protocols and potential publication bias may impact generalizability. Future research should focus on large-scale, high-quality RCTs with standardized methodologies and diverse populations to confirm these findings. Integrating these non-pharmacological nursing interventions into standard care requires a balanced approach, ensuring both their proven effectiveness and practical feasibility within the healthcare system.

## Data Availability

The original contributions presented in the study are included in the article/Supplementary Material, further inquiries can be directed to the corresponding author.
